# Examination of the psychometric properties of the FORensic oUtcome Measure (FORUM): a new outcome measure for forensic mental health services

**DOI:** 10.1192/j.eurpsy.2022.1541

**Published:** 2022-09-01

**Authors:** H. Ryland, J. Cook, R. Fitzpatrick, S. Fazel

**Affiliations:** 1 University of Oxford, Department Of Psychiatry, Oxford, United Kingdom; 2 University of Oxford, Nuffield Department Of Orthopaedics, Rheumatology And Musculoskeletal Sciences, Oxford, United Kingdom; 3 University of Oxford, Nuffield Department Of Population Health, Oxford, United Kingdom

**Keywords:** forensic psychiatry, outcome measurement, Psychometrics, Quality Improvement

## Abstract

**Introduction:**

Forensic mental health services provide care to people in secure psychiatric hospitals and specialised community teams. Measuring outcomes is important to ensure such services perform optimally, however existing measures are not sufficiently comprehensive and are rarely patient reported.

**Objectives:**

To examine a novel instrument for measuring outcomes in forensic mental health services, the FORensic oUtsome Measure (FORUM), which consists of a complementary patient reported questionnaire (FORUM-P) and clinician reported questionnaire (FORUM-C).

**Methods:**

Inpatients at a forensic psychiatric service based in a regional healthcare organization in the UK completed the FORUM-P, while members of their clinical teams completed the FORUM-C. Patients and clinicians also provided feedback on the questionnaires.

**Results:**

Sixty-two patients participated with a mean age of 41.0 years (standard deviation 11.3). For internal consistency, Cronbach’s alpha for the FORUM-P was 0.87 (95% confidence interval (CI) 0.80-0.93) and for the FORUM-C was 0.93 (95% CI 0.91-0.96). For test-retest reliability the weighted kappa for the FORUM-P was 0.44 (95% CI 0.24-0.63) and for the FORUM-C was 0.78 (95% CI 0.73-0.85). For interrater reliability of the FORUM-C the Spearman correlation coefficient was 0.47 (95% CI 0.18-0.69). The FORUM-P received an average rating of 4.0 out of 5 for comprehensiveness, 4.6 for ease of use and 3.9 for relevance, while the FORUM-C received 4.1 for comprehensiveness, 4.5 for ease of use and 4.3 for relevance.

**Conclusions:**

Outcome measures in forensic mental health can be developed with good measures of reliability and validity, and can be introduced into services to monitor patient progress.

**Disclosure:**

No significant relationships.

